# Shear Wave Elastography in the Detection of Sinusoidal Obstruction Syndrome in Adult Patients Undergoing Allogenic Hematopoietic Stem Cell Transplantation

**DOI:** 10.3390/diagnostics11060928

**Published:** 2021-05-21

**Authors:** Marten Schulz, Lam Giang Vuong, Hans Peter Müller, Martin Maibier, Frank Tacke, Igor Wolfgang Blau, Alexander Wree

**Affiliations:** 1 Department of Hepatology and Gastroenterology, Charité-Universitätsmedizin Berlin, Campus Virchow-Klinikum (CVK) und Campus Charité Mitte (CCM), 13353 Berlin, Germany; martin.maibier@charite.de (M.M.); frank.tacke@charite.de (F.T.); alexander.wree@charite.de (A.W.); 2 Department of Hematology, Oncology and Tumor Immunology, Charité-Universitätsmedizin Campus Virchow-Klinikum (CVK), 13353 Berlin, Germany; lam.vuong@charite.de (L.G.V.); igor.blau@charite.de (I.W.B.); 3 Department of Radiology, Charité-Universitätsmedizin Berlin, Campus Charité Mitte (CCM), 10117 Berlin, Germany; hans_peter.mueller@charite.de

**Keywords:** sinusoidal obstruction syndrome (SOS), veno-occlusive disease (VOD), hematopoietic stem cell transplantation (HSCT), shear wave elastography (SWE), liver stiffness measurement (LSM)

## Abstract

Hepatic sinusoidal obstruction syndrome (SOS), also known as veno-occlusive disease (VOD) can be a life-threatening complication after hematopoietic stem cell transplantation (HSCT). Diagnosis is often difficult and traditionally based on clinical parameters. Shear wave elastography (SWE) is a modern non-invasive liver stiffness measurement technique using ultrasound. In this monocentric study, we evaluated the role of SWE in diagnosing SOS/VOD in 63 adult patients undergoing HSCT from February 2020 to August 2020 in real world settings. Three patients developed SOS/VOD. This was accompanied by an increase in shear wave velocity in all three patients, indicating that this method may contribute to establishing the diagnosis SOS/VOD after HSCT.

## 1. Introduction

Sinusoidal obstruction syndrome, also known as veno-occlusive disease (SOS/VOD) is a rare, potentially life-threatening microcirculatory disorder of the hepatic sinuses that can occur after hematopoietic stem cell transplantation (HSCT). Its incidence has been reported to be as high as 60% with a mean incidence of 13.7%. Treatment regimens that have been associated with SOS/VOD contain amongst others busulfan, carmustine or cyclophosphamide [1].

In the current understanding of the disease development, a chemotherapy dependent toxic damage to hepatic sinusoidal endothelial cells leads to a local inflammation and migration of blood cells into the space of Disse conducting an obstruction of the sinusoidal microcirculation that can lead to fibrosis. This cascade promotes liver dysfunction, rising portal blood pressure and fluid retention [2,3]. Since the primary damage occurs in the hepatic sinusoids, the term “SOS” is preferred rather than “VOD” [4].

In most cases, clinical signs appear in the first month after HSCT, displaying a broad spectrum of possible symptoms from asymptomatic courses to severe cases with multi-organ failure (MOF) and mortality up to 80% [1].

In the absence of specific biomarkers, diagnosis is often difficult and is traditionally based on clinical parameters. The so-called Baltimore [5] and Seattle [6] criteria have been replaced by the revised criteria for the diagnosis of SOS/VOD proposed by the European Society for Blood and Marrow Transplantation (EBMT) [7]: in the first 21 days after HSCT, (classical) SOS/VOD is diagnosed by bilirubin ≥2 mg/dL and two of the following criteria: painful hepatomegaly, weight gain >5% and ascites. Beyond day 21 after HSCT, it is diagnosed as classical SOS/VOD or histologically proven or by the presence of two or more of the following criteria: bilirubin ≥2 mg/dL, painful hepatomegaly, weight gain >5%, ascites and hemodynamical or/and US evidence of SOS/VOD.

In order to establish a diagnosis as accurately and early as possible, imaging, especially ultrasound-based, is often beneficial. Traditional B-mode and Doppler signs such as ascites, gallbladder wall-thickening, hepatosplenomegaly, reduced or reversed portal vein flow or elevated resistive index (RI) of the hepatic artery (≥0.75) are unspecific [8,9]. Modern ultrasound-based techniques such as contrast-enhanced ultrasound (CEUS) or noninvasive liver-stiffness measurement (LSM) have shown few but promising results in diagnosing SOS/VOD [8,10]. Liver stiffness measurement is a well-established technique in detecting and grading liver fibrosis in chronic liver diseases such as viral hepatitis or non-alcoholic fatty liver disease (NAFLD) [11]. Liver stiffness can be accurately measured by shear wave elastography (SWE) of the elastic properties of liver tissue. Recently, a new technique, which additionally analyses the dispersion of shear waves has been developed as a tool to assess liver viscosity as a surrogate for inflammation [12]. The first clinical studies suggest that via shear wave dispersion imaging (SWD), necroinflammation of the liver can be measured [13,14].

In animal models, elevated stiffness parameters in SOS/VOD were measured using LSM-techniques [15,16]. Furthermore, small numbers of SOS/VOD patients have been described that were examined by different LSM methods. Fontanilla et al. reported two SOS/VOD patients whose high acoustic radiation force impulse (ARFI) elastography velocities decreased under SOS/VOD treatment [17]. Increased values in ARFI-measurements have been associated with severe complications after HSCT [18]. In addition, anecdotal reports indicate that elevated liver stiffness can be found in patients with SOS/VOD using LSM techniques such as transient elastography (TE) [19] or two-dimensional shear wave elastography (2D-SWE) [20,21] which is a method that allows the display of real-time images of shear wave propagation in a focused area in which a region of interest (ROI) can be placed for quantitative measurement. Given these scarce but promising results, we aimed at further assessing 2D-SWE in the context of diagnosing SOS/VOD in adults as a non-invasive, easily repeatable tool in clinically applicable real world settings of a high-volume tertiary care center.

## 2. Materials and Methods

In this monocentric prospective study, 63 consecutive adult patients undergoing 2D-SWE examination before allogeneic HSCT in the Department of Hematology, Oncology and Tumor Immunology, Charité-Universitätsmedizin Berlin, Germany, were included from February 2020 until August 2020. Prior to starting the conditioning regimen, all patients underwent an abdominal ultrasound (US) including grayscale US, Doppler measurement of the portal vein and 2D-SWE using Canon (former Toshiba) Aplio 500 US system (Canon Medical systems Corporation, Otawara, Tochigi, Japan). All US examinations were performed by an experienced sonographer with experience in liver ultrasound and sonoelastography (>6000 US, >2000 SWEs). All patients gave written informed consent.

The 2D-SWE value was defined as the median value of at least 3, preferably 5 to 10 SWE measurements of good quality. Real time imaging allowed the examiner to assess reliability of a measurement immediately: After homogenous color filling of the 2D-SWE elastogram and the display of parallel lines in the propagation mode, a ROI was placed at least 1 cm below the liver capsule and 3–5 cm from the transducer in a right intercostal position. Measurements were performed during a transient breath hold (without deep inspiration) avoiding large vessels and ascites according to the guidelines of the European Federation of Societies for Ultrasound in Medicine and Biology (EFSUMB) [22].

From the beginning of the conditioning regimen, laboratory tests were assessed daily in addition to daily clinical assessment for the presence of SOS/VOD until 30 days after HSCT. SOS/VOD diagnosis was established by clinical signs according to the revised criteria for the diagnosis of SOS/VOD by the EBMT [7].

In patients with clinical signs for SOS/VOD, grayscale and Doppler ultrasound and 2D-SWE were analyzed. Other possible hepatobiliary complications after HSCT were also evaluated. Follow-up ultrasound and 2D-SWE examinations were conducted if possible according to the patient’s clinical status and course. All patients with clinically established SOS/VOD diagnosis underwent an additional single SWD examination using Canon Aplio i800 ultrasound system (Canon Medical systems Corporation, Otawara, Tochigi, Japan). A total of 6–7 SWD measurements were performed as described above for SWE measurements.

Data collection and statistical analysis was performed using IBM SPSS Statistics for Windows, (Version 27.0. Armonk, NY, USA: IBM Corp). Clinical values were retrospectively collected from the patients’ medical records and entered into SPSS data sheet.

## 3. Results

### 3.1. Patient Characteristics

During the study period, 63 patients were enrolled before allogenic HSCT, 20 (31.7%) were female and 43 were male (68.3%), mean age was 54.6 (median 57) years (range 22–72 years). The three patients who developed SOS/VOD after HSCT were 36, 45 and 64 years old. The patient characteristics are displayed in Table 1 and Table 2. The most frequent underlying disease overall was acute myeloid leukemia (AML) (28 patients, 44.4%), followed by 13 patients with myelodysplastic syndrome (MDS), seven patients had lymphoma, eight patients had acute lymphatic leukemia (ALL), three patients had chronic myeloid leukemia (CML), one patient had multiple myeloma (MM), one patient had lymphomatoid granulomatosis, one patient had essential thrombocytosis (ET) and one patient had hypereosinophilic syndrome. Three patients (4.8%) developed clinical SOS/VOD (according to [7]), their underlying diseases were AML, CML and MM. As Table 2 shows, SOS/VOD was diagnosed on day +18, +18 and +21 after HSCT. 47 (74.6%) patients of our cohort had a matched unrelated stem cell donor, eight (12.7%) a matched related donor and eight (12.7%) had a mismatch donor. Two of the SOS/VOD patients had a matched unrelated donor and one had a mismatch donor.

### 3.2. Conditioning Regimen

In the study collective, 20 (31.7%) patients received either a myeloablative conditioning (MAC) with 4 × 3.2 mg/kg busulfan and 2 × 60 mg/kg cyclophosphamide or a MAC with 8–12 Gy total body irradiation (TBI) and cyclophophosphamide or fludarabine. One patient with multiple myeloma received treosulfan, thiotepa and fludarabine (TTF) as MAC. Reduced conditioning regimen (RIC) was applied for 43 patients with 3 × 12 g/m^2^ treosulfan and 5 × 30 mg/m^2^ fludarabine or 2 × 3.2 mg/kg busulfan and 5 × 30 mg/m^2^ fludarabine. All three of the SOS/VOD-patients received MAC.

### 3.3. Pretherapeutic SWE Measurement

Mean SWE at baseline before starting of the conditioning was 1.42 m/S (6.1 kPa), median SWE 1.38 m/S (5.7 kPa) in the study cohort. One patient with ALL displayed advanced liver stiffness (12.9 m/S) but did not develop clinical SOS/VOD after HSCT. SWE measurement in patients who did not develop SOS/VOD after HSCT did not indicate increased liver stiffness in this cohort: 1.42 m/S (6.1 kPa). In patients that developed SOS/VOD after HSCT, pre-transplant SWE measurements were also without signs of elevated liver stiffness: 1.29 m/S (4.9 kPa). None of the patients had ascites at baseline.

### 3.4. Pretherapeutic Portal Vein Velocity

Mean PV velocity was measured in 57 patients at baseline. Overall PV velocity at baseline was 22.5 cm/S. In patients who did not develop SOS/VOD, PV velocity was 22.4 cm/S and in patients who developed SOS/VOD baseline PV velocity was 24.7 cm/S respectively.

### 3.5. Ultrasound Findings in SOS/VOD Patients

**Figure 1:**
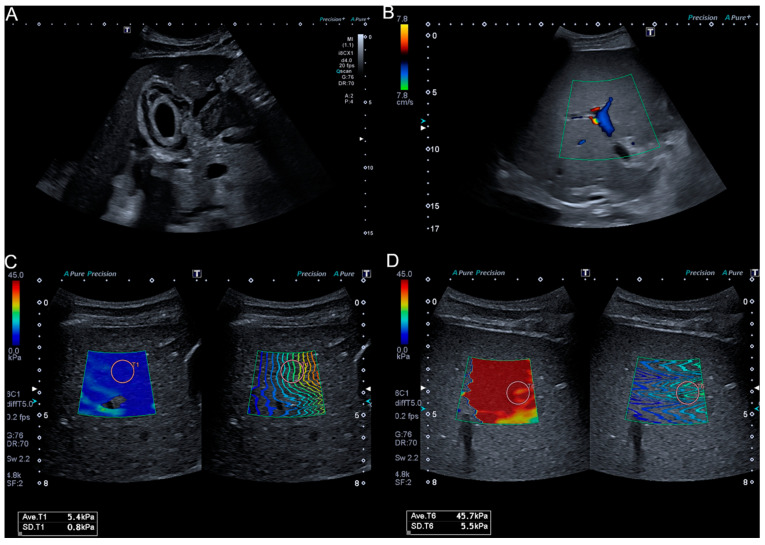
US imaging and SWE in a 36-year-old SOS/VOD patient. Gallbladder wall-thickening (**A**); reversed portal vein flow in color Doppler ultrasound (**B**); 2D-SWE before HSCT (**C**); and after development of SOS/VOD after HSCT (**D**).

The three patients who developed SOS/VOD after HSCT were clinically diagnosed by fulfilling EBMT criteria on day +18, +18 and +21 after transplantation, and all 3 patients were categorized as severe. Ultrasound after HSCT displayed signs of SOS/VOD in two patients (Patients 1 and 3) who presented a gallbladder wall-thickening, ascites and a reduced or reversed portal vein flow (as depicted for Patient 1 in Figure 1). Ascites quantities were low and there was no depiction of ascites in the region of 2D-SWE measurement. Follow-up US under defibrotide therapy displayed normalization of gallbladder wall and portal vein flow in Patient 1, and Patient 3 did not show changes in US findings under therapy. Patient 2 did not develop US signs of SOS/VOD in the course of disease.

### 3.6. SWE Measurement in SOS/VOD Patients

**Figure 2:**
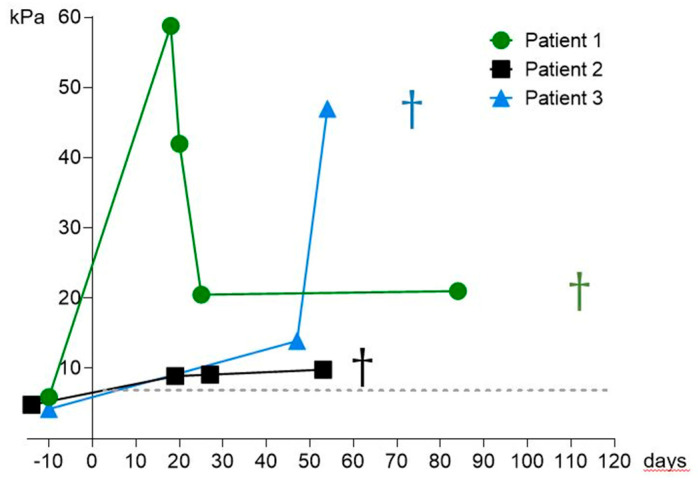
Courses of SWE values (kPa) of the three SOS/VOD patients. Day 0 is the day of HSCT, Patient 1 died on day +110, Patient 2 died on day +63, and Patient 3 on day +78.

SWE examination showed increased SWE values in all SOS/VOD patients as depicted in Figure 2 and in Table 2. Patient 1 had an increase in elasticity from 5.9 kPa to up to 58.8 kPa, Patient 2 increased from 4.7 kPa to 9.8 kPa and Patient 3 increased from 4.2 kPa before HSCT to a measured maximum SWE value of 45.5 kPa. Follow-up SWE measurements are presented in Figure 2.

### 3.7. SWE Measurement under Treatment with Defibrotide

Patient 1 had a strong early increase in SWE velocity of 2.75 m/S up to 4.17 m/S. Under defibrotide treatment, SWE velocities decreased and stabilized around 2.5 m/S (representative SWE values of Patient 1 are depicted in Figure 1). Patient 1 died on day +110. Follow-up SWE measurements in Patients 2 and 3 did not decrease in the course of disease. Patient 2 displayed constantly elevated SWE velocities between 1.7 and 1.8 m/S. SWE levels further increased in patient 3 up to 3.72 m/S. These patients died on day +63 and day +78.

### 3.8. SWD Measurement

SWD examination was performed in all three SOS/VOD patients, timepoints after HSCT were day +20, +54, +46. Mean SWD was 18.3 (m/S)/kHz, SWD values were elevated in all three patients: in Patient 1, SWD was 16.7 (m/S)/kHz, in Patient 2 SWD was 17.2 (m/S)/kHz and in Patient 3, SWD was (20.9 m/S)/kHz (the threshold for “severe” provided by the manufacturer is 16 (m/S)/kHz).

## 4. Discussion

This study aimed at further assessing the role of modern ultrasound-based techniques in the detection of SOS/VOD after HSCT in real world settings. Briefly, 2D-SWE is a method that has the advantage of visualizing the tissue at the same time of performing stiffness measurement. To our knowledge, this is the first study evaluating the role of 2D-SWE in a larger cohort of adults in the context of SOS/VOD after HSCT. Recent reports have demonstrated the usefulness of LSM by TE in adults and 2D-SWE in children in contributing to an early diagnosis of SOS/VOD after HSCT [19,20]. Recently, Colecchia and colleagues found an increase in liver stiffness values in all four patients that developed SOS/VOD in a study of 78 patients undergoing HSCT and prior LSM via TE [19] indicating that LSM can play a role in the detection of SOS/VOD. Since TE does not provide visualization of the examined tissue, results can be influenced by factors such as ascites, which is not uncommon in SOS/VOD patients and was also seen in two of our patients. By real-time visualization of the area in which LSM is performed, 2D-SWE can exclude the presence of ascites in the region of measurement resulting in reliable values. In our view, this is a clear advantage of 2D-SWE over TE in the context of SOS/VOD. Recently, Reddivalla et al. examined 25 pediatric patients before and after HSCT with 2D-SWE. All 5 patients who developed SOS/VOD displayed increased liver stiffness [20]. Lazzari and colleagues reported monitoring of defibrotide treatment using 2D-SWE in one patient with SOS/VOD after HSCT [21]. However, there was no baseline liver stiffness measured impeding conclusions on the properties of 2D-SWE in contributing to the diagnosis of SOS/VOD. Given the rarity of this potentially life-threatening disease, reported cases are few in numbers. Both Redivalla et al. and Colecchia et al. performed numerous liver stiffness measurements in all patients after HSCT. This is especially important in order to find the most accurate time point for LSM in early detection of SOS/VOD, which might even be earlier than clinical suspicion for SOS/VOD. In routine clinical practice, US and LSM examinations in the extremely vulnerable patient group soon after HSCT require more effort and time than regular examinations. Since US machines with SWE capacities are rarely exclusively utilized in transplant wards, they need to be transported there for the examination in appropriate hygiene conditions. Therefore, in our view a realistic approach to the utilization of SWE in the context of SOS/VOD is rather in single or few examinations in addition to a clinical suspicion that is not fully determined and in evaluation of clinical course and treatment response. In our cohort, all three SOS/VOD patients had a severe course of disease with MOF, all patients died despite therapy with defibrotide. SWE-measurements after HSCT were only performed if patients were stable enough to be transported, which in our opinion is a real-life approach to this clinical situation. All three SOS/VOD patients clearly displayed considerable increases in SWE values once clinical diagnosis was established compared to values before HSCT as depicted in Figure 2 and Table 2. The increase in stiffness in Patients 1 and 3 even surpassed 40 kPa (the rise of stiffness values in Patient 1 is also depicted in Figure 1). This underlines that an increase in SWE values can be a useful marker in establishing the diagnosis of SOS/VOD after HSCT. In Patient 2, no traditionally known US signs of SOS/VOD such as ascites, gallbladder wall-thickening or reduced/reversed portal vein flow could be detected but the patient displayed an increase in SWE velocity. This indicates that SWE measurement is possibly a more sensitive method for SOS/VOD detection than conventional US. Fontanilla et al. described a normalization of elevated ARFI values in two SOS/VOD patients corresponding to their treatment response [17]. In our cohort, Patients 2 and 3 did not recover under treatment and died. Their SWE values did not decrease in the course of disease. Patient 1 stabilized clinically under defibrotide treatment but remained severely compromised with high levels of bilirubin (>20 mg/dL). Accordingly, US signs of SOS/VOD such as gallbladder thickening and reversed PV flow could not be detected in follow-up US. As depicted in Figure 2, his elasticity parameters decreased and stabilized to a lower, still elevated level around 20 kPa. In our view, this finding corresponds to a (partial) treatment response. However, SWE values in our SOS/VOD patients reflected their clinical state underlining that SWE values and clinical course of SOS/VOD are associated.

A limitation of SWE in the context of SOS/VOD is the frequent occurrence of ascites that may interfere with measurements. In order to obtain reliable SWE measurements, large amounts of ascites should be drained if present. However, the possibility of visualization of ascites in SWE-measurements is a clear advantage over TE in SOS/VOD patients. Other limitations of our study are the small size of the cohort from only one center. No SWE measurements were performed in patients after HSCT without clinical signs of SOS/VOD hence evidence for earlier signs of disease that may be displayed in SWE could not be provided. Once the diagnosis SOS/VOD was established, US examinations and SWE/SWD were not performed on certain fixed time points because patients had to be stable enough to be transported which makes comparison more difficult.

Since early pathophysiological changes include migration of red blood cells, leucocytes and cellular debris into the space of Disse, leading to obstruction of the sinusoidal microcirculation [23], one can speculate that especially in these early stages that involve necroinflammation, SWD might be a helpful tool for earlier detection than traditional means of diagnosis. Even though SWD is not a widely established method, and therefore, no validated thresholds exist, our SOS/VOD patients all displayed high dispersion values compared with reported values for NAFLD or liver transplant rejection [13,14]. SWD examination in our cohort took place after clinical diagnosis was established, in two patients late in the course of disease, hence the time period when an increase of dispersion levels occurred remains unknown. This study is the first to address the modern method of SWD in the context of SOS/VOD and suggests that SWD might have a role in detecting of SOS/VOD, further research is needed and should be focused on early stages after HSCT. In our view, the combination of SWE and SWD has the potential of providing helpful information for early detection and evaluation of the clinical course of SOS/VOD after HSCT.

## 5. Conclusions

Our study provides further evidence that SWE can play an important role in the accurate non-invasive detection of SOS/VOD after HSCT in clinical routine. More research with larger cohorts is needed to further assess the role of SWE in early detection of SOS/VOD in order to initiate treatment as early as possible. SWD should also be considered in such trials.

## Figures and Tables

**Table 1:** Patient characteristics.

		Study Cohort (*n* = 63)	No SOS/VOD (*n* = 60)	SOS/VOD (*n* = 3)
disease type	AML	28 (44.4%)	27	1
	MDS	13 (20.6%)	13	0
	lymphoma	7 (11.1%)	7	0
	ALL	8 (12.7%)	6	0
	CML	3 (4.8%)	2	1
	lymphomatoid granulomatosis	1 (1.6%)	1	0
	hypereosinophilic syndrome	1 (1.6%)	1	0
	MM	1 (1.6%)	0	1
	ET	1 (1.6%)	1	0
stem cell donor	matched unrelated	47 (74.6%)	45	2
	matched related	8 (12.7%)	8	0
	mismatch	8 (12.7%)	6	1
conditioning regimen	MAC	20 (31.7%)	17	3
	RIC	43 (68.3%)	43	0
gender	female	20 (31.7%)	19	1
	male	43 (68.3%)	41	2
age (years)		55	54.9	48.3
baseline SWE (m/S)		1.42	1.43	1.29
baseline SWE (kPa)		6.1	6.2	4.9
baseline PV velocity (cm/S)		22.5 (*n* = 57)	22.4	24.7

Abbreviations: AML acute myeloid leukemia; MDS myelodysplastic syndrome; ALL acute lymphoblastic leukemia; CML chronic myeloid leukemia; MM multiple myeloma; ET essential thrombocytosis; SWE shear wave elastography; PV portal vein; MAC myeloablative conditioning with busulfan 4 × 3.2 mg/kg and 2× cyclophosphamide 60 mg/kg for AML, CML and MDS, 12 Gy and 2 × 60 mg/kg cyclophosphamide i.v. or 8 Gy and 4 × 30 mg/m^2^ fludarabine for ALL, TTF (3 × 12 g/m^2^ treosulfan, 5 × 30 mg/m^2^ fludarabine and 2.5 mg/kg thiotepa) for multiple myeloma; RIC = reduced conditioning with 3 × 12 g/m^2^ treosulfan and 5 × 30 mg/m^2^ fludarabine or 5 × 30 mg/m^2^ fludarabine and 2 × 3.2 mg/kg busulfan i.v.

**Table 2:** Characteristics of SOS/VOD patients.

Patient	Gender	Age	HSCT Type	Conditioning Regimen	Underlying Disease	Baseline 2D-SWE (m/S)	Baseline 2D-SWE (kPa)	Baseline PV Velocity (cm/S)	Clinical SOS/VOD Diagnosis †	Serum Bilirubin >2 mg/dL at Diagnosis	SWE at SOS/VOD Diagnosis (m/S)	SWE at SOS/VOD Diagnosis (kPa)	Max. Increase in SWE (m/S; kPa)	Max. SWE (m/S; kPa)	Ascites in US	Decreased/Reversed PV Flow	Gallbladder Wall Thickening	Date of US/SWE	SOS/VOD Severity †	Max. Bilirubin (mg/dL)	SOS/VOD Therapy	Outcome	SWD ((m/S)/kHz); (day)
1	male	36	allogenic (MMUD)	4 × 3.2 mg/kg busulfan, 2 × 60 mg/kg cyclophosphamide	AML	1.42	5.9	29	+18	3.26	4.17	58.8	2.75; 52.9	4.17; 58.8	yes ‡	yes	yes	+18	severe	34.71	DEF, diuretics	Death (+110)	16.7 (+20)
2	female	64	allogenic (MUD)	treosulfan, fludarabine, thiothepa	MM	1.26	4.7	29	+18	5.62	1.71	8.9	0.52; 5	1.78; 9.8	no	no	no	+19	severe	20.76	DEF, diuretics	death (+63)	17.2 (+54)
3	male	45	allogenic (MUD)	4 × 3.2 mg/kg busulfan, 2 × 60 mg/kg cyclophosphamide	CML	1.20	4.2	16	+21	4.46	2.11	13.9	2.52; 41.3	3.72; 45.5	yes ‡	yes	yes	+47	severe	35.83	DEF, diuretics	death (+78)	20.9 (+47)

Abbreviations: MUD, matched unrelated donor; MMUD, mismatch unrelated donor; AML, acute myeloid leukemia; MM, multiple myeloma; PV, portal vein; † according to Mohty et al. 2016 [7]; US, ultrasound; DEF, defibrotide; SWE, shear wave elastography; SWD, shear wave dispersion. ‡ Small amounts of ascites, no detection of ascites in the area of 2D-SWE measurement.

## Data Availability

The data are not publicly available due to privacy restrictions.
